# Effects of patch size and basal area on avian taxonomic and functional diversity in pine forests: Implication for the influence of habitat quality on the species–area relationship

**DOI:** 10.1002/ece3.4208

**Published:** 2018-06-11

**Authors:** Myung‐Bok Lee, John P. Carroll

**Affiliations:** ^1^ Daniel B. Warnell School of Forest and Natural Resources University of Georgia Athens Georgia; ^2^ School of Natural Resources University of Nebraska‐Lincoln Lincoln Nebraska

**Keywords:** diversity–area relationship, habitat preference, habitat structure, pine plantation, trait

## Abstract

Relationships between avian diversity and habitat area are assumed to be positive; however, often little attention has given to how these relationships can be influenced by the habitat structure or quality. In addition, other components of biodiversity, such as functional diversity, are often overlooked in assessing habitat patch value. In the Sandhills Ecoregion of Georgia, USA, we investigated the relationship between avian species richness and functional diversity, forest basal area, and patch size in pine forests using basal area as a surrogate for overstory structure which in turn impacts vegetation structure and determines habitat quality within a patch. We conducted bird surveys in planted mature pine stands, during breeding season of 2011. We used three classes of stand basal area (BA): OS, overstocked (BA ≥ 23 m^2^/ha); FS, fully/densely stocked (13.8 m^2^/ha ≤ BA < 23 m^2^/ha); and MS, moderately stocked (2.3 m^2^/ha ≤ BA < 13.8 m^2^/ha). MS patches showed more structural diversity due to higher herbaceous vegetation cover than other two pine stocking classes of patches. Total species richness and functional richness increased with the size of MS patches, whereas functional divergence decreased with the size of OS patches (*p *< 0.05). Functional richness tended to be lower than expected as the size of OS patches increased. Greater richness of pine–grassland species was also found at MS patches. Percent cover of MS patches within a landscape influenced positively the richness of pine–grassland species (*p *< 0.05). Our results suggest that (a) avian species–habitat area relationship can be affected by habitat quality (structural diversity) and varies depending on diversity indices considered, and (b) it is important to maintain moderate or low levels of pine basal area and to preserve large‐sized patches of the level of basal area to enhance both taxonomic and functional diversity in managed pine forests.

## 1 INTRODUCTION

Planted pine forests comprise the dominant forest type in the Southeastern United States. Although most planted pine forests are managed for commercial wood production, there have been increasing efforts to manage the forests to enhance avian diversity, especially on public lands (e.g., military bases), retaining some forest remnants where timber production is not the primary objective. A great number of studies have explored how pine patch or stand characteristics, such as age and vegetation or habitat structure within a patch, influence avian taxonomic diversity (mostly species richness) and abundance and how different management practices affect those characteristics (Dickson, Thompson, Conner, & Franzreb,^1993^; Sallabanks & Arnett, 2005; Wilson & Watts, 2000). Among the characteristics, habitat structural diversity within a pine patch has been known to strongly affect avian species. Basal area is considered one of main factors determining structural diversity, primarily but not entirely by influencing the amount of canopy cover (Melchiors, 1991). Practices such as spacing (at the stage of planting) and thinning, which primarily aim to create and maintain appropriate basal area, have been common in forest management for wildlife (Dickson et al., 1993; Melchiors, 1991). High basal area results in closed canopy, reduces light penetration, increases competition among understory plants, lowers herbaceous vegetation, and slows the growth of trees (Allen, Bernal, & Moulton, 1996; Melchiors, 1991). It can simplify habitat structure (i.e., lower structural diversity) and thus reduce overall habitat quality, especially for species preferring open forests such as early successional species, shrubland species, or pine–grassland species. Conversely, too low basal area of a patch (e.g., heavy thinning) can have a negative impact on tree nesting species and mature forest or forest interior species that prefer relatively dense canopy cover. These negative or positive effects of basal area on diversity and occurrence of avian species are often observed in hardwood forests or mixed pine–hardwood forests (Canterbury, Martin, Petit, Petit, & Bradford, 2000; McDermott & Wood, 2011; Wang, Lesak, Felix, & Chweitzer, 2006). Although several studies have been conducted to determine the effects of basal area on avian species in other ecoregions (Wilson, Masters, & Bukenhofer, 1995; Wood, Burger, Bowman, & Hardy, 2004), little is known about how basal area influences avian diversity in the Sandhills Ecoregion.

The species–area relationship or the diversity–area relationship is widely discussed in ecology for decades although it has been rarely explored in southern pine forests. Positive relationship between species richness or abundance and the size of habitat patch (here, patch is defined as “a surface area that differs from its surroundings in nature or appearance”; Turner & Gardner, 2015) has been well documented in other systems (Arrhenius, 1921; MacArthur & Wilson, 1963; Rosenzweig, 1995 for review; Hill & Curran, 2003; Lindenmayer & Fischer, 2006). However, it has been debated that the main factor influencing species richness may not be area per se, but habitat diversity, which is often highly correlated with area (Boecklen, 1986; Shochat, Abramsky, & Pinshow, 2001). The area per se hypothesis expects the positive relationship due to sampling effect (e.g., the larger area would be sampled more and thus more individuals and species would be detected) and due to reduction in extinction risk and increase in immigration. The habitat diversity or habitat heterogeneity hypothesis assumes that as area increases, the number of different habitats, which could be used by different species, increases and so does species richness. A number of recent studies indicate that these two hypotheses are not mutually exclusive and the degree of area effect can be affected by habitat diversity or habitat type (Davidar, Yoganand, & Ganesh, 2001; Kallimanis et al., 2008; Marini, Bommarco, Fontana, & Battisti, 2010; Triantis, Mylonas, Lika, & Vardinoyannis, 2003). The species–area relationship can also vary with species traits (especially, dispersal ability or mobility), matrix type (environmental features surrounding a patch), fragmentation, connectivity, and so on (Freeman, Oliver, & van Aarde, 2018; Marini et al., 2010; Scheffer et al., 2006). However, it remains speculative how habitat structure or habitat quality affects the species–area relationship in birds (Blake & Karr, 1987). Unlike natural forests, most planted mature pine forests maintain relatively uniform conditions across a patch because they are planted with single tree species and managed at the patch or stand level. Thus, managed pine forests provide a good opportunity to explore the relationship between patch size (area) and avian diversity by reducing the confounding effects from variations in habitat diversity correlated with area.

Taxonomic biodiversity, especially species richness, is commonly used as a surrogate for biodiversity in ecological studies. However, there is a growing consensus that inferences solely based on taxonomic diversity can be misled. Considering other components of biodiversity such as phylogenetic, genetic, or functional diversity is critical to improve our understanding on ecological processes associated with biodiversity (Mouchet, Villéger, Mason, & Mouillot, 2010; Pavoine & Bonsall, 2011; Webb, Ackerly, McPeek, & Donoghue, 2002). As a trait‐based measure of biodiversity, functional diversity quantifies the diversity or dissimilarity in morphological, physiological, and ecological traits among species or organisms, which strongly affect ecosystem functioning (Hooper et al., 2005; Tilman, 2001). It has been applied to a wide range of ecological studies that examine community assemblage rules, relationships between biodiversity and environmental characteristics or ecosystem services, and prediction of ecosystem functioning (Cadotte, Carscadden, & Mirotchnick, 2011; Flynn et al., 2009; Gagic et al., 2015; Luck, Carter, & Smallbone, 2013; Mouillot, Graham, Villéger, Mason, & Bellwood, 2013; Petchey & Gaston, 2006). Different patterns between taxonomic and functional diversity have been also reported (Devictor et al., 2010; Lee & Martin, 2017; Murray et al., 2017), suggesting that functional diversity can convey different information about communities than taxonomic diversity and complement traditional species richness (Diaz & Cabido, 2001; Mouchet et al., 2010; Vandewalle, 2010). Several recent studies on the species–area relationship also demonstrate the importance of considering multifacets of diversity as the relationship can be inconsistent between species richness and functional diversity and even between functional diversity indices (Ding, Feeley, Wang, Pakeman, & Ding, 2013; Karadimou, Kallimanis, Tsiripidis, & Dimopoulos, 2016). In pine forests, functional diversity has been seldom incorporated in the study of biodiversity and thus little is known about the functional diversity–area relationship.

We investigated the relationship between avian diversity (species richness and functional diversity), patch size (area), and basal area in planted mature pine forests in central‐east Georgia. We used basal area as a surrogate for habitat or vegetation structure and as a measure of habitat quality within a pine patch. Our goal was to determine (a) what levels of basal area are necessary for pine forest management to conserve avian diversity in the region and (b) how patch size and basal area interplay and affect avian diversity, that is, how habitat quality represented by levels of basal area can influence the species–area relationship in birds. We expected that both taxonomic and functional diversity would decrease with increasing basal area because high basal area could reduce structural diversity of vegetation (i.e., habitat quality) within a stand, especially the amount of understory herbaceous vegetation cover by creating too dense canopy cover. We also expected that the effect of patch size on avian diversity would vary with the level of basal area, namely habitat quality of the patch.

## 2 METHODS

### 2.1 Study area

**Figure 1:**
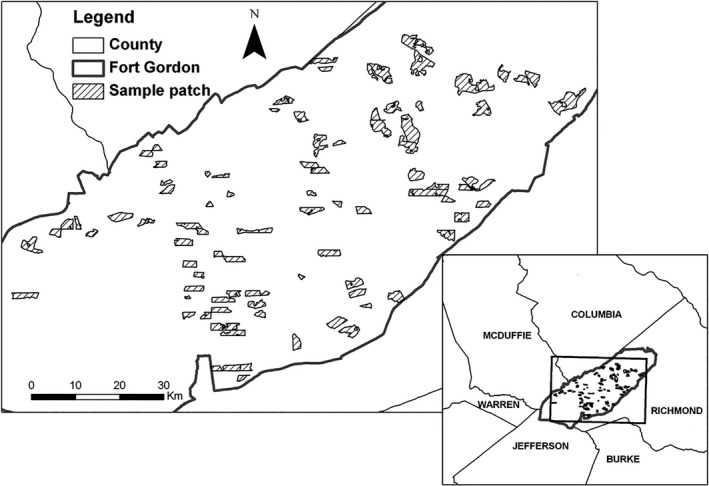
Study areas at Fort Gordon, Georgia (central‐east Georgia), and locations of sample pine patches surveyed in 2011. Sample patches included both loblolly pine patches and longleaf pine patches

Our study was conducted in pine stands (hereafter patches) in the U.S. Army Fort Gordon, Georgia (Figure 1). It is located in the Sandhills Ecoregion. Fort Gordon was established in 1917 and is 22,600 ha in size with forest comprising 80% of the land area. Large open areas are maintained for military training purposes. Pine forests are dominated by planted loblolly pine (*Pinus taeda*) throughout the study areas, and there are some patches of longleaf pine (*Pinus palustris*). Slash pines (*P. elliottii*) or shortleaf pines (*P. echinata*) are also mixed with loblolly pines in some areas. The ages of pine patches vary across our study sites; however, old pine patches (>75 years) are relatively rare and most pine patches are young (<20 years) or mid‐aged (20–75 years). Overstory and midstory of hardwood forest and mixed forest largely consist of sweetgum (*Liquidambar styraciflua*), sassafras (*Sassafras albidum*), black cherry (*Prunus serotina*), flowering dogwood (*Cornus florida*), and oak (*Quercus* spp.). Sparkleberry (*Vaccinium arboreum*) is also commonly found in the midstory. The understory is dominated by yellow jessamine (*Gelsemium sempervirens*), muscadine grapes (*Muscadinia rotundifolia*), greenbrier (*Smilax spp*.), brambles (*Rubus*spp.), blueberry (*Vaccinium* spp.), broomsedge bluestem (*Andropogon virginicus*), low panicgrass (*Dicanthelium* spp.), wiregrass (*Aristida stricta*), and lespedeza (*Lespedeza* spp.).

### 2.2 Sample patches

Using modified 2011 forest inventory data and 2009 land cover map of Fort Gordon, we selected 130 mid‐aged loblolly and longleaf pine patches to represent mature pine. Within a patch, one point was established randomly at 50‐70 m away from any edge (road, other types of vegetation or land cover, etc.). All patches were located at relatively undeveloped landscape, containing <7% of built‐up structure within a 1 km radius circle surrounding a sample point.

We defined a pine patch as a stand where vegetation composition and structure are relatively uniform. If basal area or other vegetation characteristics highly varied within a patch, we divided the patch to keep homogeneous characteristics. We delineated patch boundaries from aerial photos and ground truthing. Patch size was calculated using ArcGIS and average patch size was 13.8 ha (±10.1, standard deviation; range 2 ha–54 ha). This patch size can be small compared to the size of pine stands in timberland. However, we emphasize that a patch in our study is relatively intact and homogeneous and thus we can reduce confounding effects of potential habitat heterogeneity that often increases with patch size.

To determine basal area (BA; m^2^/ha) of sample patches and of adjacent patches, we used inventory data of Fort Gordon. The inventory data were collected using the 10 BAF variable plot method at >3 plots/stand. These data included both softwoods and hardwoods, but hardwoods were minor in our sample patches; therefore, we assumed that the BA data of Fort Gordon could represent the BA of softwoods. The inventory data grouped all stands into five BA classes. We regrouped them into three classes because two of the classes were rare: OS, overstocked (≥23 m^2^/ha); FS, dense/fully stocked (13.8 m^2^/ha ≤ BA < 23 m^2^/ha); MS, moderately/sparsely stocked (2.3 m^2^/ha ≤ BA < 13.8 m^2^/ha). We also verified the BA class of patches, particularly those selected for our study using our vegetation data collected in 2011 (Lee, 2013). There was good congruence between BA class from Fort Gordon inventory data and BA class from our vegetation survey data, confirming the accuracy of the inventory data that were used to determine the BA class of patches chosen for our study.

### 2.3 Bird surveys and vegetation surveys

We performed bird surveys three times during May–June 2011, using fixed‐radius point counts (Ralph, Geupel, Pyle, Martin, & DeSante, 1993). At each point, an observer recorded species seen or heard within a 50 m radius of a sampling point during 10‐min period. Two observers conducted the survey, and they were rotated between sites to reduce observer effects. We also alternated survey order so that three counts for each point were carried out at different start time to minimize the effect of time of day. Each survey was performed between dawn to 1100 EDT. We did not conduct surveys during inclement weather, such as high wind or rain.

To explore variation in local vegetation characteristics (percent cover of tree, shrub, and herbaceous vegetation) among BA classes, we performed vegetation sampling at each point between late June and early August in 2011. We established four 5 m radius circular plots in each cardinal direction at a fixed distance of 30 m from a sample point. Within each of the circular plots, vegetation data were collected using a protocol modified from Point Reyes Bird Observatory (PRBO) Point Count Veggie (Relevé) Protocol (http://www.prbo.org/cadc/songbird/pc/relevepr.html). Percent cover of vegetation in tree (>5 m in height), shrub (0.5–5 m in height), and herb (<0.5 m in height) layers, and on the ground were visually estimated. The values of each vegetation characteristic estimated from four circular plots were averaged to represent the value at the sample patch.

### 2.4 Taxonomic and functional diversity

We included all bird species (except flyovers, nocturnal species, and raptors) detected at least once during the survey (Supporting Information Appendix S1 for species list). We used species richness as a metric of taxonomic diversity and the maximum number of individuals observed among 3 visits as abundance. We calculated total richness, that is, the number of species detected, and the richness of pine–grassland species, which include major conservation concern species such as Bachman's sparrow (*Peucaea aestivalis*) and Northern Bobwhite (*Colinus virginianus*) in the Southeastern United States. Pine–grassland species inhabit relatively open forest with early successional or grassland‐like understory vegetation. Of 48 species, 10 species were classified into pine–grassland species (Ehrlich, Dobkin, & Wheye, 1988; Hamel, 1992; Wilson et al., 1995).

Functional diversity was represented by three indices that depict different aspects of functional diversity and are independent to each other (Mouchet et al., 2010; Schleuter, Daufresne, Massol, & Argillier, 2010; Villéger, Mason, & Mouillot, 2008). Functional richness (FRic) quantifies the volume of functional space occupied by species. Functional evenness (FEve) measures the regularity of species’ abundances in functional space. Functional divergence (FDiv) describes the distribution of abundance within functional space. We calculated these indices based on traits considered functionally important in other studies due to their association with species’ resource acquisition and use (Flynn et al., 2009; Luck, Lavorel, McIntyre, & Lumb, 2012; Luck et al., 2013; Calba, Maris, & Devictor, 2014; Supporting Information Appendix S1): body mass, food type (insects/arthropods, seeds/grains, all types [omnivorous]), foraging behavior and location (foliage gleaning, bark gleaning, ground foraging, aerial foraging), and migratory status (resident or migrant). While body mass was a continuous trait type, others were binary trait types (e.g., insects/arthropods = 1 if the main diet of species is insects and insects/arthropods = 0 otherwise). Traits of 48 species were obtained from “The Birds of North America” online database (Poole, 2005) and Ehrlich et al. (1988) and from Dunning (2008) for missing body mass data. We computed functional diversity indices using dbFD function in the FD package (Laliberté, Legendre, & Shipley, 2014) in R 3.4.1 (R Core Team, 2017), which created the Gower dissimilarity matrix from a trait matrix of 48 species, performed a principal coordinate analysis (PCoA) with the distance matrix, and used the first 4 PCoA axes as new traits to estimate the values of functional diversity indices. The number of PCoA axes characterizes the quality of functional space and thus significantly affects the measurement of functional diversity. To evaluate the quality of functional space determined by those 4 PCoA axes, we calculated the mean squared deviation (mSD; Maire, Grenouillet, Brosse, & Villéger, 2015). When mSD value is close to 0, the quality of functional space is considered high. The mSD of the first 4 PCoA axes (0.0028) was lower than the mSD of other PCoA axes (0.0034 ‐ 0.0079), confirming that 4 PCoA axes chosen for our study were appropriate.

Among functional diversity indices, FRic was strongly correlated with total species richness (Pearson's correlation *r* = 0.81, *p* < 0.001). We adopted a null model approach to assess whether changes in observed FRic were independent of changes in species richness. We generated 999 communities by randomly choosing species from the species pool (48 species detected across all sample points) without replacement and by randomly assigning the species to each sample point but maintaining the species richness as constant within a point. Following the approach of Gotelli and Rohde (2002), we calculated the standardized effect size (SES.FRic) for each sample patch, which measures the deviation in observed FRic from expected FRic: SES.FRic = (Observed FRic − mean expected FRic)/standard deviation of expected FRic. Expected FRic values were calculated from 999 random communities. Randomization was performed using the picante package (Kembel et al., 2010) in R 3.4.1.

### 2.5 Analysis

Although all sample patches were located at relatively undisturbed sites, in order to minimize potential matrix effects from other types of land cover, we excluded points if (a) percent cover of pine forest was <50%, and (b) percent cover of any open space and/or disturbed lands was >20% within a 1 km radius circle of the sample point. We calculated the relative proportion of land cover using FRAGSTATS 3.3 (McGarigal, Cushman, Neel, & Ene, 2002). A total of 85 points was selected for final analysis: OS, *n* = 20; FS, *n* = 41; MS, *n* = 24. For analysis, we did not distinguish pine types (longleaf vs. loblolly pine), because our previous study showed no significant difference in vegetation characteristics and in avian species richness between two pine types (Lee, 2013).

To determine how patch size and basal area (i.e., habitat quality/structure) affect avian diversity, we constructed a regression model by including an interaction between patch size and basal area and log‐transformed percent cover of MS stands within a 1 km radius area surrounding a sample point (logMS) as explanatory variables. We added logMS to the model to take into account differences in matrix quality (basal area) surrounding a sample patch. Within a 1 km radius area, percent cover of MS was negatively correlated with percent cover of FS (*r* = −0.534, *p* < 0.001) and of OS (*r* = −0.605, *p* < 0.001), but there was no significant correlation between percent cover of FS and OS. Thus, we chose percent cover of MS and normalized using a log‐transformation. We also log‐transformed patch size and total richness, and log(*x* + 1)‐transformed the richness of pine–grassland species. If we did not find a significant interaction effect in the model, we reran the model without the interaction. In addition, we performed Kruskal–Wallis test to compare percent vegetation cover at tree, shrub, and herb layers, as well as percent ground vegetation cover among three BA classes. This test was also performed to verify whether BA can properly describe variations in habitat condition. We also determined whether SES.FRic value of each patch differed from zero, that is, whether the observed FRic value significantly differed from the expected value (mean value of 999 random communities). If SES.FRic was outside 95% confidence interval (CI) under the normal distribution, the value was considered to be significant at *α* = 0.05.

In addition, we examined the presence of spatial correlation in our data using a Moran's I test. For the indices where spatial autocorrelation was detected, we adopted spatial autoregressive modeling (SAR), especially spatial lag model and spatial error model (Kissling & Carl, 2008). We chose the SAR model that produced the lower Akaike information criterion (AIC) value (Burnham & Anderson, 2002) and used the SAR model to make inferences. We also performed Levene's test to assess the homogeneity of variance assumption. All models satisfied the assumption (*p* > 0.1 in all cases). SAR modeling was carried out in R 3.4.1, using “spdep” package (Bivand, Hauke, & Kossowski, 2013).

## 3 RESULTS

**Figure 2:**
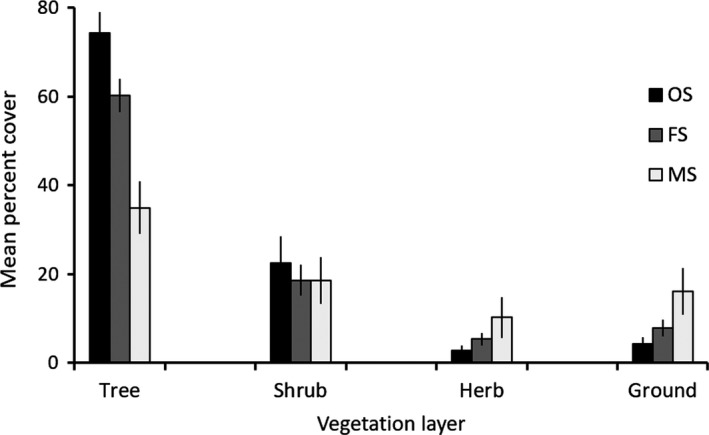
Mean and 95% confidence interval (CI) of vegetation cover among three basal area classes in pine forest in Georgia, USA. The vertical line on a bar represents 95% CI. At each vegetation layer, if 95% CIs did not overlap, the vegetation cover was considered significantly different between basal area classes. Abbreviations: OS, overstocked (BA ≥ 23 m^2^/ha, *n* = 20); FS, fully/densely stocked (13.8 m^2^/ha ≤ BA < 23 m^2^/ha, *n* = 41); MS, moderately stocked (2.3 m^2^/ha ≤ BA < 13.8 m^2^/ha, *n* = 24)

Among the three basal area classes evaluated, mean percent cover of vegetation at all layers except shrub layer significantly differed (Figure 2): tree layer, Kruskal–Wallis *χ* ^2^ = 48.55, *p* < 0.001; herb layer, Kruskal–Wallis *χ* ^2^ = 10.06, *p* = 0.007; ground vegetation cover, Kruskal–Wallis *χ* ^2^ = 16.19, *p* < 0.001. Mean percent cover of grasses and forbs on the ground was also different between all pairs of BA classes given nonoverlapped 95% CIs. Vegetation cover at tree layer was highest at OS patches; however, herbaceous vegetation (grasses and forbs), which was the dominant vegetation cover at herb layer and on the ground, was lowest at OS patches but highest at MS patches (Figure 2). These patterns suggest that BA can be an appropriate surrogate representing variations in vegetation or habitat structure and thus habitat quality within a patch in our study sites.

**Figure 3:**
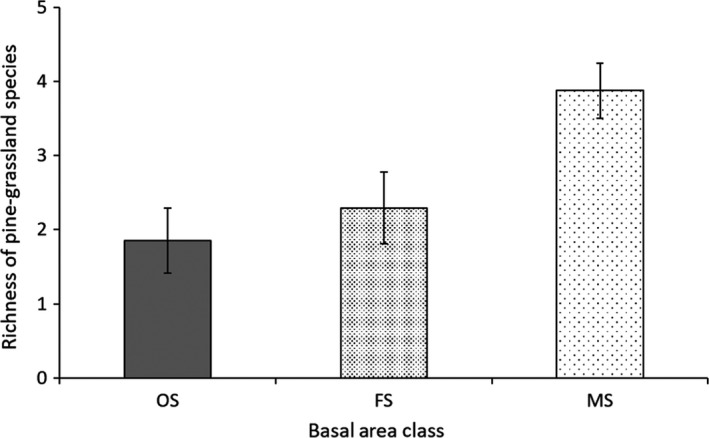
Relationship between basal area classes and the richness of pine–grassland species. The vertical line on a bar represents 95% confidence interval (CI). If 95% CIs of the classes did not overlap, they were considered significantly different. Abbreviations: OS, overstocked (BA ≥ 23 m^2^/ha, *n* = 20); FS, fully/densely stocked (13.8 m^2^/ha ≤ BA < 23 m^2^/ha, *n* = 41); MS, moderately stocked (2.3 m^2^/ha ≤ BA < 13.8 m^2^/ha, *n* = 24)

The richness of pine–grassland species responded significantly to basal area (Table 1): greater values at MS patches compared to OS or FS patches, but no significant difference between OS and FS patches (Figure 3). The richness of pine grassland species also increased as the percent cover of MS stands increased within a landscape (Table 1). There was no interaction effect between patch size and basal area class on this response variable.

**Table 1:** Model results summarizing effects of basal area class (OS, overstocked; FS, fully/densely stocked; MS, moderately stocked), patch size (SIZE), and percent cover of MS stands within a landscape (logMS) on taxonomic and functional avian diversity in pine forests in Georgia, USA. In all models, OS was set as a reference. Thus, all estimates were compared to OS except SIZE in a model without an interaction between patch size and basal area class and logMS. Significant estimates were in bold (*p *< 0.05)

Response variable	Explanatory variable						
	Intercept	SIZE^a^	FS	MS	logMS	SIZE × FS	SIZE × MS
Total richness^a^,^b^	**2.208**	−0.06	−0.234	−0.41	0.036	0.153	**0.293**
Pine–grassland^a^,^b^	**0.671**	0.041	0.054	**0.321**	**0.120**		
FRic^c^	0.225	−0.062	−0.211	−0.282	0.015	0.112	**0.185**
SES.FRic	0.719	−0.526	−**0.166**	−1.202	0.097	**0.718**	0.642
FEve	**0.765**	−0.006	0.009	0.010	−0.003		
FDiv	**0.911**	−**0.039**	−**0.107**	−0.089	0.004	**0.046**	0.043

a Log transformed.

b Spatial autocorrelation; spatial error model.

c Spatial autocorrelation; spatial lag model.

**Figure 4:**
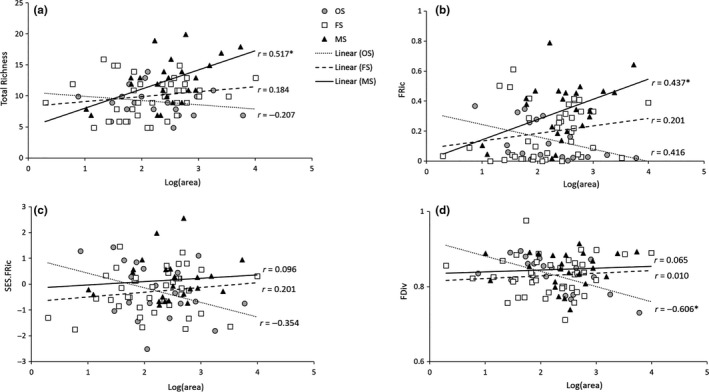
Regression plot of total richness (the number of species; a), functional richness (FRic; b), standardized effect size of functional richness (SES.FRic; c), and functional divergence (FDiv; d) with patch size at each of three basal area classes. “*r*” and “*” indicate the Pearson's correlation value and the significance at α = 0.05, respectively. Abbreviations: OS, overstocked (BA ≥ 23 m^2^/ha, *n* = 20); FS, fully/densely stocked (13.8 m^2^/ha ≤ BA < 23 m^2^/ha, *n* = 41; MS, moderately stocked (2.3 m^2^/ha ≤ BA < 13.8 m^2^/ha, *n* = 24)

Total species richness, FRic, SES.FRic, and FDiv showed that the effect of patch size could vary depending on basal area classes, namely, habitat structure or habitat quality (Table 1 and Figure 4). Total richness and FRic increased with the size of MS patches, but tended to decrease as the size of OS patches increased, resulting in significant differences in the regression slope between MS and OS patches. The correlation between patch size and these two variables was also significant at MS patches. While SES.FRic showed similar patterns, a difference in the regression slope was found between OS and FS patches. All SES.FRic values of FS patches fell between −1.96 and 1.96, indicating that SES.FRic did not differ from 0 and FRic was neither higher nor lower than expected at FS parches (Figure 4). Although similar results were observed in other basal area classes, there were several significant cases: FRic was significantly higher than expected at two MS patches and lower than expected at one OS patch.

Relatively steep decline in FDiv was also found at OS patches: negative correlation between FDiv and patch size (Figure 4). While FDiv did not show clear patterns with increasing the size of FS or MS patches, the regression slope of OS patches was significantly different from the slope of FS patches (Table 1). Unlike other diversity indices, FEve did not show significant responses to any explanatory variables (Table 1).

## 4 DISCUSSION

Our results demonstrated that habitat quality of a pine patch, which was based on structural diversity represented by the level of basal area, can influence the relationship between avian diversity (both taxonomic and functional diversity) and patch size (area) in pine forests. We did not find a strong effect of patch size without accounting for variations in habitat quality within a patch. Although there were variations in the significance of responses among diversity indices, communities at large‐sized pine patches with moderate or low level of basal area (MS patches) were composed of more species and more functionally unique species than communities at other levels of basal area. Conversely, dissimilarity in functional traits between abundant species and other species decreased with increasing the size of patches with high level of basal area (OS patches). Basal area and the amount of MS patches, that is, the amount of a good quality of habitat, within a landscape also affected avian diversity, especially the richness of pine–grassland species: greater richness at MS patches and at landscapes with high percent cover of MS stands.

### 4.1 Effects of basal area and patch size on taxonomic diversity

Relationship between basal area and avian species richness can vary depending on species or a group of species of interest; however, a negative effect of high level of basal area has often been documented in other studies. For instance, Canterbury et al. (2000) found that the richness of shrubland species was strongly negatively correlated with tree basal area. Wang et al. (2006) reported that in oak–hickory forest, Red‐eyed Vireo (*Vireo olivaceus*; forest interior) was most abundant at closed canopy (control and 25% basal area removal plots), whereas Indigo Bunting (*Passerina cyanea*; classified as early successional species in their study) was most abundant at open canopy (≥50% basal area removal plots). Some early successional species such as Blue Grosbeak (*Passerina caerulea*) and Prairie Warbler (*Setophaga discolor*) were observed only at open canopy sites. Similar responses of some of the species were described in the research that compared species abundance or richness among thinned plots and unthinned plots (Garrison, 1986; Kerpez & Stauffer, 1989). In our study, those early successional or shrubland species were pine–grassland species. Consistent with the findings of other studies, we found low richness of pine–grassland species at OS patches and low total richness at large‐sized OS patches, but great richness of pine–grassland species at MS patches.

Although high basal area is detrimental to species inhabiting open forest, too low basal area (e.g., clear‐cut stand or a heavily thinned stand) could also reduce species richness, especially by negatively affecting forest interior species or species preferring dense canopy cover. Overall richness may be great at the level between the two extremes (i.e., intermediate or relatively low level of basal area). McDermott and Wood (2011) described that during the postbreeding period, richness and abundance of late successional (mature forest) species were lower in clear‐cut stands than in hardwood stands of two classes of basal area (2.0–3.7 m^2^/ha and 5.3–7.0 m^2^/ha), although the difference was not statistically significant. Wang et al. (2006) also found the highest territory density and species richness at intermediate open canopy (50% and 75% basal area removal plots, respectively). Wang et al. (2006) did not clearly describe the levels of basal area. The range of basal area used to define basal area classes in their study could be different from ours. However, given the similar responses of pine–grassland species between our study and Wang et al.'s study (2006), MS is likely to be the intermediate open canopy. In addition, although the level of basal area classified as MS was broader than the basal area used in McDermott and Wood's study (2011), the two classes of basal area were the lower limit of MS and thus they could be classified as MS. In particular, we also observed the richness of forest interior species did not differ among three basal area classes, which indicates that MS is not as low as to affect forest interior species negatively (Lee, 2013). This supports that MS is neither high nor too low basal area, representing the intermediate level of basal area. Moreover, it should be noted that MS patches showed relatively diverse habitat structure by maintaining higher amount of herbaceous vegetation cover than others, especially OS patches. This suggests that OS and MS patches can represent low and high quality of habitats, respectively. FS (fully/densely stocked) may be considered as moderate quality habitat in our systems.

Numerous studies have described the positive relationship between patch size and avian species richness (Bellamy, Hinsley, & Newton, 1996; Blake & Karr, 1987; McIntype, 1995; Turner, Gerwin, & Lancia, 2002; Yamaura, Kawahara, Iida, & Ozaki, 2008). In particular, a significant effect of patch size is often observed in habitat specialists (Matthews, Eden Cottee‐Jones, & Whittaker, 2014). Blake and Karr (1987) and McIntype (1995) compared species richness and composition among different sizes of woodlots in an agricultural matrix: habitat generalists (Blake & Karr, 1987) and edge species (McIntype, 1995) were dominant in smaller woodlots, but forest interior species were more abundant in larger woodlots. Other studies also observed a positive effect of patch size on the richness of other habitat specialists such as shrubland or woodland birds (Ambuel & Temple, 1983; Huth & Possingham, 2011; Lehnen & Rodewald, 2009; Rodewald & Vitz, 2005). A large patch is likely to contain more interior zones that reduce negative edge effects than a small patch, and thus it can provide the species with more areas unaffected by disturbance (Baker, 1992; Harris, 1984; Pickett & Thompson, 1978). Unlike these studies, we did not find a significant effect of patch size on the richness of pine–grassland species that are habitat specialists and include species of conservation concern sensitive to disturbance or avoid edge zones. However, pine–grassland species showed significantly positive responses to increasing percent cover of MS stands within a landscape (Table 1). There was no correlation between MS patch size and the percent cover of MS stands within a landscape. This indicates that pine–grassland species can be more influenced by the amount of good quality of habitats than the size of the habitat per se within the landscape scale we considered. Slightly inconsistent results between our study and others may be related to variations in matrix surrounding a patch. Some of previous studies were performed in agricultural‐dominant matrix, which was very contrast to a woodlot. Conversely, all of patches in our study were surrounded by other pine stands, which can be less inhospitable compared to agricultural lands.

### 4.2 Effects of basal area and patch size on functional diversity

Among three functional diversity indices, FRic showed similar patterns observed in total species richness: a positive response to increasing the size of MS patches compared to that of OS patches. That is, a bird community at MS patches was composed of more unique species than a community at OS patches, particularly when the patch size was large. However, regardless of the level of basal area, FRic was positively correlated with species richness as reviewed in other studies (Mouchet et al., 2010; Schleuter et al., 2010). This indicates that variations in FRic may be associated with changes in species richness. However, SES.FRic showed a significant difference in regression slope between FS patches and OS patches and the tendency of decreasing SES.FRic with increasing the size of OS patches, suggesting that the interaction between patch size and basal area has an impact on functional richness independent of changes in species richness. It is also noteworthy that although most SES.FRic values did not differ from 0, when SES.FRic value was significant, it was positive at MS patches (2 of 24 patches) and negative at OS patches (1 of 20 patches). In addition, more SES.FRic values tended to be far below 0 at OS patches, but above 0 at MS patches. The difference between the observed values of functional diversity index and the expected values of the index is often used to explore the relative role of environmental filtering and limiting similarity (niche complementarity) in determining community assemblages (Mouchet et al., 2010; Swenson, 2014). Negative SES values (i.e., values below 0) are considered as an evidence of dominant role of environmental filtering, whereas positive SES values support the important role of limiting similarity. In our study, moderate or low level of basal area (MS) could provide different resources or habitats for birds by forming heterogeneous vegetation structure, particularly increasing vegetation cover at herb layer and on the ground, which will increase a chance for functionally dissimilar species to coexist. On the contrary, high basal area (OS) may homogenize the habitat structure within a patch and thus narrow the range of traits that persists in the environment, which in turn decrease functional dissimilarity among coexisting species.

FEve depicts the evenness of species’ abundances in functional space. FEve increases as species with different traits are equally distributed in functional space and abundances among those species are identical (Mason, Mouillot, Lee, & Wilson, 2005; Mouchet et al., 2010; Schleuter et al., 2010). It is also used to estimate whether resources are under‐ or over‐utilized, which influences productivity and susceptibility to invasion (Mason et al., 2005; Schleuter et al., 2010). Unlike other indices, FEve was not affected by any variables, suggesting that the level of basal area and the patch size do not have an impact on resource utilizations.

While FDiv did not show a significant response at MS patches, as patch size increased, FDiv was significantly low at OS patches, especially compared to FS patches. FDiv is high when abundant species is far from the center of functional space and low when it is close to the center (Mason et al., 2005; Schleuter et al., 2010). FDiv measures the degree of niche differentiation. A high value indicates high niche differentiation, namely, high dissimilarity between the most abundant species and other species and thus low resource competition between them (Mason et al., 2005; Mouchet et al., 2010). As a result, resource use can be more efficient and ecosystem functioning may be enhanced in communities with high FDiv (Mason et al., 2005). Our results show that at large‐sized OS patches, abundant species are more closely located to the center of functional space and their traits are similar, which indicate low efficiency in resource use.

### 4.3 Species–area relationship and effects of habitat quality in pine forests

While the species–area relationship is often assumed to be positive in ecological studies, there has been a long debate on the relative importance of area per se and habitat diversity. Findings from several studies suggest that even if area may be a stronger positive factor, habitat diversity can change the slope of the positive relationship, for example, fast species accumulation with increasing area as habitat diversity increased (Kallimanis et al., 2008). However, most research has focused on the number of different habitats (largely composition) as a measure of habitat diversity. Few studies have explored the combined effects of area and habitat quality or habitat structural diversity on avian species richness, particularly in pine forests. Huth and Possingham (2011) modeled woodland bird species–area relationships by incorporating vegetation structural diversity. They found a more significant effect of patch size at high habitat structural diversity (i.e., high‐quality habitat) than at low habitat structural diversity (i.e., low quality habitat). Our results were similar to their findings and partly consistent with the result of Kallimanis et al. (2008). As patch size increased, total richness in MS patches significantly increased, but total richness in OS patches tended to decrease. Therefore, the slope of species–area regression line was affected by habitat quality of a patch. This pattern implies that area may not be a main factor influencing avian species richness in pine forests. Rather, its effect could be indirect and intercorrelated with habitat quality. This is also somewhat congruent with the finding of Triantis et al. (2006) who described the similar species–area–habitat diversity (the number of habitats) relationship at small scale, which was associated with the small island effect, that is, no significant effect of area on species richness below a certain island (or patch) size threshold.

The species–area relationship can exhibit diverse patterns when species’ trait (including functional diversity, functional/ecological guilds), evolutionary lineage (e.g., phylogenetic diversity), or species mobility is considered (Bell, Phillips, Nielsen, & Spence, 2017; Davidar et al., 2001; Ding et al., 2013; Karadimou et al., 2016; Marini et al., 2010; Mazel et al., 2014). For instance, Karadimou et al. (2016) showed that the species–area relationship could be positive, neutral, or even negative depending on the indices of functional diversity in plants. Mazel et al. (2014) also found that phylogenetic and functional diversity of mammals reached their maximum values more quickly than species richness with increasing area although overall patterns were similar. Marini et al. (2010) described a relatively strong effect of habitat diversity on species richness than area and mobility in orthopteran species, whereas Bell et al. (2017) reported more sensitive responses of ground beetle species with large body size and low dispersal ability to changes in area. These diverse patterns suggest that it is important to approach the species–area or diversity–area relationship from multiperspectives by taking into account different aspects of biodiversity and potential environmental factors that may influence the relationship.

Likewise, our study considered species richness, functional diversity, and ecological guild (pine–grassland species), and included habitat quality as well as potential effects of matrix surrounding a pine patch in our analysis. In particular, to our knowledge, there are no studies that examined the relationship between avian functional diversity, area, and habitat quality in pine forests. Functional diversity showed diverse relationships depending on the indices, which was similar to previous studies focused on the functional diversity–area relationship (Ding et al., 2013; Karadimou et al., 2016). Although the functional diversity–area relationship was vague at FS patches, there was a significant pattern at MS and OS patches: increasing FRic at MS patches but decreasing FDiv at OS patches with increasing their patch size. FRic and SES.FRic also tended to decrease as the size of OS patches increased. These patterns confirm the indirect effect of area through habitat quality on avian diversity in pine forests.

Although the findings of our study provide insights on how habitat quality affects the species–area relationship in pine forests and how we can manage pine forests for avian diversity conservation, there are some aspects we could not consider and need further investigations. We did not account for the spatial arrangement of patches, especially the connectivity among patches, and possible variations in landscape characteristics at a larger scale. Considering the effects of the percent cover of MS stands within a landscape, smaller‐sized patches can still play an important role in the conservation of avian species when connectivity is high. However, if land cover or other habitat types which were minor in our study become dominant at a larger landscape scale, they may influence the avian community in the patch. In that case, the size of patch in inhospitable matrix should be larger than the size in a favorable matrix because adverse edge effects will penetrate further into the patch in an inhospitable matrix. Moreover, patch shape affects the amount of the edge of the patch. As complexity of patch shape increases, the amount of edge increases. More complex shapes are often observed at larger patches (e.g., Ewers & Didham, 2007; Krummel, Gardner, O'Neill, & Coleman, 1987). There is a trade‐off between shape complexity and patch size. As patch shape is often relatively uniform in most planted pine forests, we assumed the patch shape would not significantly affect our results. However, the information about the relationship between patch shape and proper patch size could be valuable in developing better forest management plans.

## 5 MANAGEMENT IMPLICATIONS

Our findings provide valuable information for future forest management at Fort Gordon and mature pine forest dominant landscapes, especially in the Sandhills Ecoregion. Our results suggest that moderate or low level of basal area needs to be maintained to improve avian diversity. Both taxonomic and functional diversity can be promoted by increasing the size of a patch containing moderate or low levels of basal area (MS). The richness of pine–grassland species that include conservation concern species in the Southeastern United States is more likely to be enhanced by maintaining the basal area of pine patch to moderate or low levels or increasing the amount of MS stands within a landscape. Given that the levels of basal area of most stands at Fort Gordon are overstocked (OS) or fully/densely stocked (FS), we first recommend improving habitat quality by reducing basal area to moderate or low levels. It is also important to preserve large MS patches and to increase the connectivity among small MS patches within a landscape. For future study, we suggest exploring how patch size interacts with matrix characteristics, habitat connectivity, and patch shape. It will provide crucial information on determining the optimal or minimum patch size required for avian conservation in this region.

## CONFLICT OF INTEREST

None declared.

## AUTHORS CONTRIBUTION

M‐BL and JPC conceived the project and interpreted the results. M‐BL designed the study, conducted field surveys, analyzed the data, and wrote the manuscript. JPC provided editorial assistance.
