# Supplementary material for: Effects of patch size and basal area on avian taxonomic and functional diversity in pine forests: Implication for the influence of habitat quality on the species–area relationship
Source: Ecol Evol. 2018 Jun 11;8(14):6909–20. doi: 10.1002/ece3.4208 (PMC6065337; doi:10.1002/ece3.4208)
Supplement: Supplementary file 1 [file ECE3-8-6909-s001.docx]

Appendix S1. List of species and trait used for analysis. Abbreviation: FBM, body mass; IN, insects/arthropods; GR, seeds/grains; AL, all diet types (omnivorous); FG, foliage gleaning; BG, bark gleaning; GR, ground foraging; AE, aerial foraging; Migratory, migratory status.

| Common name | Scientific name |  | Diet |  |  | Foraging | |  |  |  |
| --- | --- | --- | --- | --- | --- | --- | --- | --- | --- | --- |
|  |  | FBM | IN | GR | AL | FG | BG | GR | AE | Migratory |
| Acadian Flycatcher | *Empidonax virescens* | 12.8 | 1 | 0 | 0 | 0 | 0 | 0 | 1 | 1 |
| American Crow | *Corvus brachyrhynchos* | 467 | 0 | 0 | 1 | 0 | 0 | 1 | 0 | 0 |
| American Goldfinch | *Spinus tristis* | 12.5 | 0 | 1 | 0 | 1 | 0 | 0 | 0 | 0 |
| American Kestrel | *Spinus tristis* | 120 | 1 | 0 | 0 | 0 | 0 | 0 | 1 | 0 |
| American Robin | *Turdus migratorius* | 77.3 | 1 | 0 | 0 | 0 | 0 | 1 | 0 | 0 |
| Bachman's Sparrow^1^ | *Aimophila aestivalis* | 18.4 | 0 | 1 | 0 | 0 | 0 | 1 | 0 | 0 |
| Black-and-white Warbler | *Mniotilta varia* | 11 | 1 | 0 | 0 | 0 | 1 | 0 | 0 | 1 |
| Blue-gray Gnatcatcher | *Polioptila caerulea* | 6 | 1 | 0 | 0 | 1 | 0 | 0 | 0 | 0 |
| Brown-headed Cowbird^2^ | *Molothrus ater* | 48.9 | 1 | 1 | 0 | 0 | 0 | 1 | 0 | 0 |
| Brown-headed Nuthatch^1^ | *Sitta pusilla* | 10.4 | 1 | 0 | 0 | 0 | 1 | 0 | 0 | 0 |
| Blue Grosbeak^2^ | *Passerina caerulea* | 27.5 | 1 | 1 | 0 | 0 | 0 | 1 | 0 | 1 |
| Blue Jay | *Cyanocitta cristata* | 70.53 | 0 | 0 | 1 | 0 | 0 | 1 | 1 | 0 |
| Brown Thrasher | *Toxostoma rufum* | 68.8 | 0 | 0 | 1 | 0 | 0 | 1 | 0 | 0 |
| Carolina Chickadee | *Poecile carolinensis* | 10.8 | 1 | 0 | 0 | 1 | 0 | 0 | 0 | 0 |
| Carolina Wren | *Thryothorus ludovicianus* | 18.6 | 1 | 0 | 0 | 1 | 0 | 1 | 0 | 0 |
| Chipping Sparrow^1,3^ | *Spizella passerina* | 12.3 | 0 | 1 | 0 | 0 | 0 | 1 | 0 | 1 |
| Common Grackle | *Quiscalus quiscula* | 92.2 | 0 | 0 | 1 | 0 | 0 | 1 | 0 | 0 |
| Downy Woodpecker | *Picoides pubescens* | 21.3 | 1 | 0 | 0 | 0 | 1 | 0 | 0 | 0 |
| Eastern Bluebird | *Sialia sialis* | 30.5 | 1 | 0 | 0 | 0 | 0 | 0 | 1 | 0 |
| Eastern Towhee | *Pipilo erythrophthalmus* | 39.3 | 0 | 0 | 1 | 0 | 0 | 1 | 0 | 0 |
| Eastern Wood-Pewee^1^ | *Contopus virens* | 14.1 | 1 | 0 | 0 | 0 | 0 | 0 | 1 | 1 |
| Fish Crow | *Corvus ossifragus* | 270 | 0 | 0 | 1 | 0 | 0 | 1 | 0 | 0 |
| Great Crested Flycatcher | *Myiarchus crinitus* | 33.5 | 1 | 0 | 0 | 0 | 0 | 0 | 1 | 1 |
| Hairy Woodpecker | *Picoides villosus* | 50.15 | 1 | 0 | 0 | 0 | 1 | 0 | 0 | 0 |
| Indigo Bunting^1^ | *Passerina cyanea* | 14.38 | 1 | 0 | 0 | 1 | 0 | 1 | 0 | 1 |
| Kentucky Warbler | *Oporornis formosus* | 14.7 | 1 | 0 | 0 | 1 | 0 | 1 | 0 | 1 |
| Mourning Dove | *Zenaida macroura* | 123 | 0 | 1 | 0 | 0 | 0 | 1 | 0 | 0 |
| Northern Bobwhite^1^ | *Colinus virginianus* | 170 | 0 | 1 | 0 | 0 | 0 | 1 | 0 | 0 |
| Northern Cardinal^4^ | *Cardinalis cardinalis* | 43 | 1 | 1 | 0 | 0 | 0 | 1 | 0 | 0 |
| Northern Flicker | *Colaptes auratus* | 129 | 1 | 0 | 0 | 0 | 0 | 1 | 0 | 0 |
| Northern Mockingbird | *Mimus polyglottos* | 47.2 | 1 | 0 | 0 | 0 | 0 | 1 | 0 | 0 |
| Northern Parula | *Parula americana* | 7.6 | 1 | 0 | 0 | 1 | 0 | 0 | 0 | 1 |
| Ovenbird | *Seiurus aurocapilla* | 21.7 | 1 | 0 | 0 | 0 | 0 | 1 | 0 | 1 |
| Pine Warbler^1^ | *Dendroica pinus* | 11.9 | 1 | 0 | 0 | 1 | 1 | 0 | 0 | 0 |
| Pileated Woodpecker | *Dryocopus pileatus* | 234 | 1 | 0 | 0 | 0 | 1 | 0 | 0 | 0 |
| Prairie Warble^1,3^ | *Dendroica discolor* | 7.7 | 1 | 0 | 0 | 1 | 0 | 0 | 0 | 1 |
| Purple Martin | *Progne subis* | 48.8 | 1 | 0 | 0 | 0 | 0 | 0 | 1 | 1 |
| Red-bellied Woodpecker | *Melanerpes carolinus* | 57.6 | 1 | 0 | 0 | 0 | 1 | 0 | 0 | 0 |
| Red-eyed Vireo | *Vireo olivaceus* | 19.9 | 1 | 0 | 0 | 1 | 0 | 0 | 0 | 1 |
| Red-headed Woodpecker^1,5^ | *Melanerpes erythrocephalus* | 71.6 | 0 | 0 | 1 | 0 | 1 | 0 | 1 | 1 |
| Summer Tanager | *Piranga rubra* | 28.87 | 1 | 0 | 0 | 1 | 0 | 0 | 1 | 1 |
| Tufted Titmouse | *Baeolophus bicolor* | 20.5 | 1 | 0 | 0 | 1 | 1 | 0 | 0 | 0 |
| White-eyed Vireo | *Vireo griseus* | 11.7 | 1 | 0 | 0 | 1 | 0 | 0 | 0 | 1 |
| Worm-eating Warbler | *Helmitheros vermivorum* | 15.2 | 1 | 0 | 0 | 1 | 0 | 0 | 0 | 1 |
| Wood Thrush | *Hylocichla mustelina* | 50.1 | 1 | 0 | 0 | 0 | 0 | 1 | 0 | 1 |
| Yellow-billed Cuckoo | *Coccyzus americanus* | 62.6 | 1 | 0 | 0 | 1 | 0 | 0 | 0 | 1 |
| Yellow-throated Vireo | *Vireo flavifrons* | 18 | 1 | 0 | 0 | 1 | 0 | 0 | 0 | 1 |
| Yellow Warbler | *Dendroica petechia* | 9.6 | 1 | 0 | 0 | 1 | 0 | 0 | 0 | 1 |

^1^Pine-grassland species

^2^Insects are more often consumed than seeds/grains (and fruits).

^3^Partial migrants: this species can also be considered as resident species.

^4^Seeds/grains (and fruits) are more often consumed than insects.

^5^Aerial foraging is more often used compared to bark gleaning.
